# Stimulants

**Published:** 1856-08

**Authors:** 


### STIMULANTS,

WHETHER of wine, or ale, or opium, or alcohol, are the greatest enemies of our kind. It is a wide mistake that the lower classes mainly fall into habits of intoxication; the very brightest minds of the past age and of our own time have been prematurely wiped out by the stealthy fiend *Alcohol.* Of the stars of a preceding century, which have gone out in the night of drink, to shine no more, we might name Addison, and Steele, and Moreland, and Sheridan, and Charles Lamb, and Theodore Hook, with myriads of others. And of our own time, what a long array, which delicacy to the living forbids us to marshal by name, of all professions and of every calling! And in addition, not a few of the daughters of our land fall, unsuspected, into the arms of the remorseless destroyer.

We are not opposed to the moderate, the rational use of tea or coffee, for these and other beverages may be advantageously employed. Against the immoderate use of so called "stimulants," whether in the milder forms of beers, wines and cordials, or of those more decidedly alcoholic, there are two infallible safeguards—one for a sage, one for a simpleton. For the latter, for the overwhelming majority, there is only one ground of safety, and it may be thus plainly stated:—

If you never touch a drop of any preparation containing alcohol, you will most assuredly never die in the gutter; if you ever do touch a drop, you may.

There is no middle ground which any man or woman can safely tread, only that of total and most uncompromising abstinence.

To the very few who are wisely firm, who have that strength of character which is the parent of the most perfect self control, we may give a safe advice. Use a specified amount at specified times, and never, under any circumstances, without medical advice, or under great urgency, increase that amount by a single drop in quantity or in frequency. And after all, to be perfectly safe—

"Touch Not—Taste Not—Handle Not."

